# Nanometric MIL-125-NH_2_ Metal–Organic Framework as a Potential Nerve Agent Antidote Carrier

**DOI:** 10.3390/nano7100321

**Published:** 2017-10-12

**Authors:** Sérgio M. F. Vilela, Pablo Salcedo-Abraira, Isabelle Colinet, Fabrice Salles, Martijn C. de Koning, Marloes J. A. Joosen, Christian Serre, Patricia Horcajada

**Affiliations:** 1 APMU, IMDEA Energy Institute, Avda. Ramón de la Sagra 3, E-28935 Móstoles, Madrid, Spain; sergio.vilela@imdea.org (S.M.F.V.); pablo.salcedo@imdea.org (P.S.-A.); 2 Institut Lavoisier de Versailles, Université de Versailles St Quentin, UMR CNRS 8180, 45 Avenue des Etats-Unis, University Paris Saclay, 78035 Versailles, France; icolinet@gmail.com (I.C.); christian.serre@ens.fr (C.S.); 3 Institut Charles Gerhardt Montpellier UMR 5253 CNRS UM, Université Montpellier, Place E. Bataillon, 34095 Montpellier CEDEX 05, France; fabrice.salles@umontpellier.fr; 4 TNO, Lange Kleiweg 137, NL-2288GJ Rijswijk, The Netherlands; m.dekoning@tno.nl (M.C.d.K.); marloes.joosen@tno.nl (M.J.A.J.); 5 Institut des Matériaux Poreux de Paris, Ecole Normale Supérieure, Ecole Supérieure de Physique et de Chimie Industrielles de Paris, FRE CNRS 2000, PSL Research University, Paris 75005, France

**Keywords:** MIL-125-NH_2_, metal–organic frameworks, nanoparticles, colloidal stability, drug delivery, pralidoxime

## Abstract

The three-dimensional (3D) microporous titanium aminoterephthalate MIL-125-NH_2_ (MIL: Material of Institut Lavoisier) was successfully isolated as monodispersed nanoparticles, which are compatible with intravenous administration, by using a simple, safe and low-cost synthetic approach (100 °C/32 h under atmospheric pressure) so that for the first time it could be considered for encapsulation and the release of drugs. The nerve agent antidote 2-[(hydroxyimino)methyl]-1-methyl-pyridinium chloride (2-PAM or pralidoxime) was effectively encapsulated into the pores of MIL-125-NH_2_ as a result of the interactions between 2-PAM and the pore walls being mediated by π-stacking and hydrogen bonds, as deduced from infrared spectroscopy and Monte Carlo simulation studies. Finally, colloidal solutions of MIL-125-NH_2_ nanoparticles exhibited remarkable stability in different organic media, aqueous solutions at different pH and under relevant physiological conditions over time (24 h). 2-PAM was rapidly released from the pores of MIL-125-NH_2_ in vitro.

## 1. Introduction

**Figure 1:**
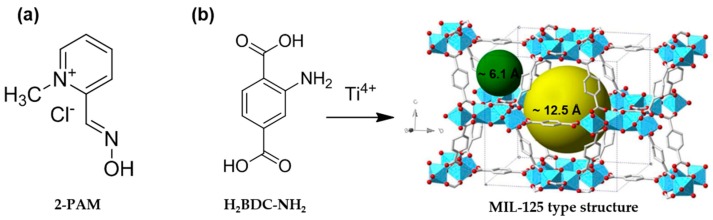
Representation of the structure of (**a**) 2-pyridinium aldoxime methyl chloride (2-PAM) and (**b**) reaction scheme for the preparation of MIL-125-NH_2_ (MIL = Material of Institut Lavoisier), highlighting its cavities of 6.1 (green sphere) and 12.5 Å (yellow sphere) from 2-aminoterephthalic acid (H_2_BDC-NH_2_) and Ti^4+^. Titanium polyhedra, oxygen and carbon are represented in blue, red and clear grey, respectively. Hydrogen atoms were omitted for the sake of clarity.

Organophosphate (OP) compounds, such as insecticides and nerve agents, are known for their ability to inhibit acetylcholinesterase (AChE) [1] by forming a covalent bond with a serine residue in the active site of AChE. AChE is an enzyme that rapidly hydrolyses the neurotransmitter acetylcholine (ACh) in cholinergic synapses in peripheral as well as in central nervous tissues [2]. Inhibition of AChE by OPs causes accumulation of ACh in the synapses and overstimulation of ACh receptors, resulting in severe symptoms such as convulsions, flaccid muscle paralysis, seizures and ultimately, death. Several efforts have been applied to the prevention and/or treatment of OP poisoning. Prophylactic strategies are mainly based on aggressive prosecution of suspected terrorists, preparedness in our communities and personal protective equipment that is able to capture and degrade the toxin. On the other hand, current therapeutic treatment of OP poisoning entails the administration of a mixture of atropine (to block the AChE receptor), diazepam (an anticonvulsant) and a compound generally referred to as “oxime”, which is capable of restoring the activity of AChE. An example of such an oxime is pralidoxime (2-pyridinium aldoxime methyl chloride or 2-PAM; Figure 1a), which reactivates OP-inhibited AChE by attacking the covalent bond between the serine and the OP [3]. Unfortunately, as a result of its permanent charge, 2-PAM penetrates the blood brain barrier (BBB) poorly, limiting its action at central nervous sites [4].

Several strategies and protocols have been developed for the adsorption and/or conjugation of pharmaceutical drugs using non-toxic nanoparticles (NPs) for the preparation of drug delivery systems [5,6,7,8,9,10,11,12,13,14]. Particularly, incorporation of 2-PAM into different nanocarriers has been proposed to overcome the previously mentioned drawback [15,16,17]. Although mesoporous silica and solid lipid NPs have shown promising results, the problem is far from being solved [18]. Apart from the potential delivery of 2-PAM into the brain, sustained release of 2-PAM into the bloodstream could lead to pharmaceutically relevant blood levels of the drug for prolonged periods of time, which would be beneficial in case of intoxication by more persistent agents such as VX (ethyl({2-[bis(propan-2-yl)amino]ethyl}sulfanyl)(methyl)phosphinate) [19].

Metal–organic frameworks (MOFs), traditionally proposed for other industrial fields (separation, catalysis, sensing, etc.) [20], appear to be promising drug nanocarriers due to their high drug loading capacities and controlled releases, associated with their exceptional porosity (up to Brunauer-Emmett-Teller surface (*S*_BET_) = 7000 m^2^ g^−1^; pore diameter = 3–98 Å), and easily tunable structure and composition [21,22]. Despite the proven interesting features of some biocompatible MOF nanocarriers in the encapsulation and release of many different therapeutic molecules (e.g., antitumorals, analgesics and biological gases) [23,24], nerve agent antidotes have never been addressed. In addition, some of these crystalline hybrid materials have recently demonstrated the ability to capture and catalytically degrade chemical warfare agents [25,26,27].

In particular, the microporous titanium aminoterephthalate MIL-125-NH_2_, first isolated by several of us [28], has not yet been proposed as a drug carrier. Nevertheless, this structure possesses physicochemical properties required in the biomedical field, specifically: (i) a significant porosity (*S*_BET_ = 1130 m^2^ g^−1^ and tetragonal or octahedral cavities of ca. 6.1 and 12.5 Å, respectively, accessible through micropores of 5–7 Å (see Figure 1b) able to host important loadings of different molecules [29,30]; (ii) very good chemical stability in water and organic solvents [31,32]; (iii) the presence of –NH_2_ moieties, able to establish hydrogen bonds with guest molecules (e.g., 2-PAM); and (iv) an a priori biocompatible character, associated with the inert biological behavior of titanium (rat oral 50% lethal dose (*LD*_50_) = 464 and > 2000 mg kg^−1^ for TiCl_4_ and TiO_2_, respectively [33,34]) and the low toxicity of the ligand (50% inhibitory concentration (IC_50_) in HeLa and J774 cells = 600 and 20 μg mL^−1^, respectively) [35]. To date, the only bio-application of MIL-125-NH_2_ was recently proposed by Wang et al. [36], who developed a MIL-125-NH_2_-hemoglobin nanoconjugate. The resulting complex was able to retain the gas-binding capacity of hemoglobin, suggesting that MIL-125-NH_2_-hemoglobin could be potentially used as an O_2_ carrier. Additionally, the visible-photocatalytic activity of MIL-125-NH_2_ [37] has been used to perform several catalytic reactions (e.g., cycloaddition of epichlorohydrin [31], Cr(VI)-Cr(III) reduction under visible light irradiation [38]), and suggested for the degradation of harmful volatile organic compounds (VOCs) [39]. 

Taking into account the above, nanometric MIL-125-NH_2_ seems to be an appropriate tool for optimizing the treatment of nerve agent poisoning by the potential VOC degradation as well as vectorization and controlled release of antidotes. Therefore, we present here the synthesis of high yields of nanometric MIL-125-NH_2_ for the encapsulation and release of the nerve agent antidote 2-PAM. Firstly, nano-sized MIL-125-NH_2_ has been prepared by optimizing the previously reported synthetic conditions. The obtained MIL-125-NH_2_ NPs were fully characterized by different standard solid state techniques (powder X-ray diffraction-PXRD, infrared spectroscopy-IR, thermogravimetric analysis-TGA, scanning electron microscopy-SEM and argon adsorption measurements, among others), paying particular attention to the evaluation of its colloidal stability under different conditions (solvent, pH and relevant physiological media). Finally, the incorporation of 2-PAM within the MIL-125-NH_2_ NPs was assessed through a joint experimental and computational approach, investigating the 2-PAM kinetics of release and colloidal stability of 2-PAM-loaded MIL-125-NH_2_ NPs under in vitro conditions.

## 2. Results and Discussion

### 2.1. Synthesis and Colloidal Stability of MIL-125-NH_2_ Nanoparticles 

Distinct synthetic methodologies have been used previously to prepare nano-scaled MIL-125-NH_2_: (i) solvothermal synthesis (150 °C/20 h or 50 °C/16 h) [40,41,42]; (ii) microwave-assisted solvothermal route (100 °C/15 min or 150 °C/1 h) [36,43]; and (iii) under reflux for 72 h [44]. Keeping in mind the desire for a simple, easy and low-cost synthetic approach, we optimized an atmospheric pressure method to obtain smaller MIL-125-NH_2_ NPs that are compatible with intravenous administration. Thus, a reactive solution composed of H_2_BDC-NH_2_ and titanium isopropoxide (TiO^i^Pr) in an appropriate mixture of solvents [*N*,*N*’-Dimethylformamide (DMF):MeOH:H_2_O = 1:0.25:0.0045 (*v*/*v*)] was prepared in a round-bottom flask and kept at 100 °C for 32 h (see Material and Methods for further details). The obtained yellow powder was centrifuged and washed with DMF and methanol. It is worth noting the efficiency of the synthetic procedure (final yield ~90%), which achieved a good space-time-yield (36.5 kg m^−3^ day^−1^) when compared with other previously reported laboratory scale-up syntheses of MOFs [45]. The nano-sized MIL-125-NH_2_ was fully characterized. Powder X-ray diffraction (PXRD) pattern (Figure S1) shows the characteristic Bragg reflections of MIL-125-NH_2_ with broader diffraction peaks, in agreement with the smaller particle size. In line with this, FEG-SEM images (Figure S2) show spherical and more or less elongated nanoparticles of 170 ± 90 nm (n = 100 particles; note here that although the definition identifies NPs as having dimensions below 100 nm, especially in the area of drug delivery, relatively large NPs are also denoted as nanoparticles instead of submicronic particles). When compared with previously synthesized MIL-125-NH_2_ also using a room pressure method (300–400 nm) [44], these particles are smaller and more monodispersed.

The TGA curve (Figure S3) shows a first weight loss of ca. 24% (from 30 to 95 °C), which can be attributed to the complete evacuation of solvent molecules retained in the pores. Above 300 °C, the combustion of the ligand gives rise to the collapse of the framework and successive formation of TiO_2_ (weight loss: obs. 37.3% vs. cal. 38.6%; calculated from the dried form of MIL-125-NH_2_). Due to the presence of solvent molecules in the pores of MIL-125-NH_2_, its Fourier transform infrared (FTIR; Figure S4) spectrum exhibits a very broad band in the 3700–2500 cm^−^^1^ range. Although it is difficult to assign all vibration modes occurring in this spectral region, the two bands centered at ca. 3458 and 3384 cm^−^^1^ which may correspond to the stretching υ_asym_(N–H) and υ_sym_(N–H) vibration bands, respectively, of the –NH_2_ groups in the MOF structure [46]. Additionally, the absence of a very intense band between 1725 and 1700 cm^−^^1^ suggests the coordination of both carboxylic acid groups, belonging to H_2_BDC-NH_2_, to Ti cations as well as the absence of free remaining ligand. 

Ar sorption measurements show a type I isotherm, characteristic of microporous materials (Figure S5). The BET surface area and micropore volume values are slightly lower than those of the bulk MIL-125-NH_2_ (1400 vs. 1890 m^2^ g^−^^1^ and 0.55 vs. 0.75 cm^3^ g^−^^1^), which could be related to the presence of an amorphous fraction. Additionally, the permanent porosity of MIL-125-NH_2_ was also confirmed through the determination of the pore size distribution, exhibiting pore diameters ranging from ca. 5 to 14 Å, in agreement with the pore sizes determined from crystallographic distances corresponding to cages of 6.1 and 12.5 Å, accessible through microporous windows of 5–7 Å (see Figure S9 in the ESI). This data was also corroborated by theoretical calculations (see Figure S10 in the ESI). 

One of the main challenges concerning MOF NPs is their colloidal stability in different media. Frequently, one is faced with several issues regarding the aggregation of these nano-sized materials, complicating their storage and their application as isolated NPs. In this investigation, the colloidal stability of the MIL-125-NH_2_ NPs in different media is described by measuring their hydrodynamic size by dynamic light scattering at room temperature (DLS; Table 1; see Material and Methods for experimental details). When suspended in DMF, methanol or ethanol, MIL-125-NH_2_ shows a small and monodispersed particle size ranging from 210 ± 100 to 320 ± 100 nm (polydispersity indexes (PdIs) from 0.06 ± 0.03 to 0.263 ± 0.003; Table 1), in agreement with previous FEG-SEM observations (170 ± 90 nm). In contrast, the MIL-125-NH_2_ aqueous solution exhibits larger particle sizes. In general, charged particles with ζ-potential above +30 mV or below −30 mV present enough electrostatic repulsion to insure colloidal stability [47]. Thus, the larger particle size of the MIL-125-NH_2_ aqueous solution can be attributed to the aggregation of the NPs as a consequence of the almost neutrally charged surface (ζ-potential = −7 ± 4 mV; note here the influence of the initial pH, see below for further explanations), which contrasts with the higher ζ-potentials measured for ethanol or methanol (from −40 to −47 mV, respectively). 

**Figure 2:**
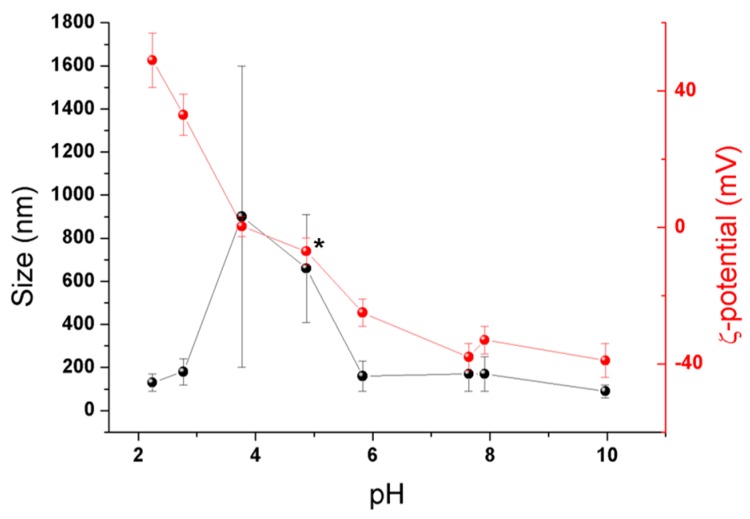
Particle size (black) and ζ-potential (red) of MIL-125-NH_2_ NPs in water as a function of pH; the autogenous pH (ca. 4.9) is identified in the plot with an asterisk (*).

In parallel to the previously described experiments, the colloidal stability of MIL-125-NH_2_ NPs was assessed as a function of the pH (Figure 2). In short, the nano-sized MIL-125-NH_2_ was dispersed in water and the pH was adjusted in the 2–10 pH range (see Materials and Methods for further details). At lower pH (<2.8), MOF NPs exhibit a positive surface charge from ca. 33 to 49 mV, ensuring enough electrostatic repulsion to avoid NPs aggregation. In contrast, the low absolute ζ-potential values of ca. 0.3 ± 3 and −7 ± 4 mV measured at pH values of ca. 3.8 and 4.9 (autogenous pH) led to larger particle sizes (ca. 800–1000 nm) and higher polydispersity (PdI) values (from 0.263 ± 0.003 to 0.320 ± 0.005), associated to an important NP aggregation. In the 5.9–10.0 pH range, the absolute ζ-potential values are higher (from −25 ± 4 to −39 ± 5 mV), maintaining the colloidal stability of the MIL-125-NH_2_ NPs. In summary, lower pH values enhance the presence of negatively charged species, while higher pHs promote the presence of positively charged ones. Considering the pKa of the carboxylic acids of the ligand (pKa ~ 3.5 and 4.4) and the amine group (pKa ~ 5.0 for the 2-aminobenzoic acid), the positive surface charge at pH < 4 might be explained by the protonated state of both amine and carboxylic groups (NH_3_^+^ and –COOH). In contrast, negatively charged surfaces at pH > 5 might be related with the presence of deprotonated carboxylates (–COO^−^) and neutral amines (–NH_2_). Finally, surface change close to the isoelectric point, at pH ~ 4–5, might be associated with the charge compensation between the negative charges of the deprotonated carboxylate groups (–COO^−^) and the positively charged protonated amines (–NH_3_^+^). Although one cannot exclude the presence of Ti–OH or Ti–OH_2_ species at the outer surface, these observations suggest that the external surface is mostly composed of organic linkers.

### 2.2. Encapsulation of 2-PAM

The nerve agent antidote 2-PAM was successfully encapsulated into the nano-sized MIL-125-NH_2_ using a simple impregnation method, by suspending the NPs in a 2-PAM methanolic solution (see Material and Methods for further details). To gain insight into the encapsulation rate and the location of 2-PAM entrapped in the pores, the resulting 2-PAM@MIL-125-NH_2_ material was characterized by several solid-state techniques. 

After 2-PAM encapsulation, the 2-PAM@MIL-125-NH_2_ NPs appeared to be highly robust without any structural modification associated with the impregnation procedure (Figure S6). The crystallinity of the material remained intact and no secondary phase (e.g., recrystallized drug) was identified.

The thermogravimetric curves of MIL-125-NH_2_ NPs before and after encapsulation have different profiles (Figure S7). After the drug adsorption, an additional weight loss below 300 °C was observed: the first one of ca. 15% corresponds to the release of solvent molecules and the second one of ca. 10.9 ± 2.2% (calculated from several thermograms acquired for the composite material), from 125 to 320 °C, was attributed to the combustion of 2-PAM molecules. Above this temperature, the collapse of the framework occurred, leading to the formation of TiO_2_. Using Grand Canonical Monte Carlo (GCMC) simulations, it was also possible to estimate the saturation capacity in 2-PAM (note here that 2-PAM might be considered combined with Cl^−^ to obtain a global neutral charge). The theoretical estimation was evaluated without taking into account the effect of the solvent and reached 30.0 wt % (i.e., almost three times the experimental value (10.9 wt %)). The discrepancy between experimental measurements and theoretical loading might be mainly related to the use of generic force fields (UFF in particular), not specifically developed for such precise applications, as well as the presence of the impregnation solvent (i.e., methanol), which competes with 2-PAM for adsorption within the MIL-125-NH_2_ pores. Although the 2-PAM loading capacity of MIL-125-NH_2_ is lower than in solid lipid nanoparticles (SLN; 30.8% *w*/*w*) and poly(lactic-co-glycolic acid) nanoparticles (PLGA; 7.1–68.8% *w*/*w*), the loading rate is in the same range than the previously reported liposome system (5.3–11.9% *w*/*w*) [16,17,18]. Nevertheless, the photocatalytic activity of MIL-125-NH_2_ could be an important advantage in simultaneously degrading nerve agents. In addition, considering this work as proof of the concept, the oximes loading capacity could be enhanced by using other MOF topologies.

Regarding its morphological features, 2-PAM@MIL-125-NH_2_ exhibits a shape and particle size totally comparable with the ones of MIL-125-NH_2_ NPs (110 ± 50 vs. 170 ± 90 nm; *n* = 100 particles; Figure S2). Similarly, the ζ-potential values are quite similar (−23 and −19 mV for MIL-125-NH_2_NPs and of 2-PAM@MIL-125-NH_2_, respectively), ruling out any significant adsorption of 2-PAM at the outer NP surface, which corroborates its successful encapsulation into the pores.

**Figure 3:**
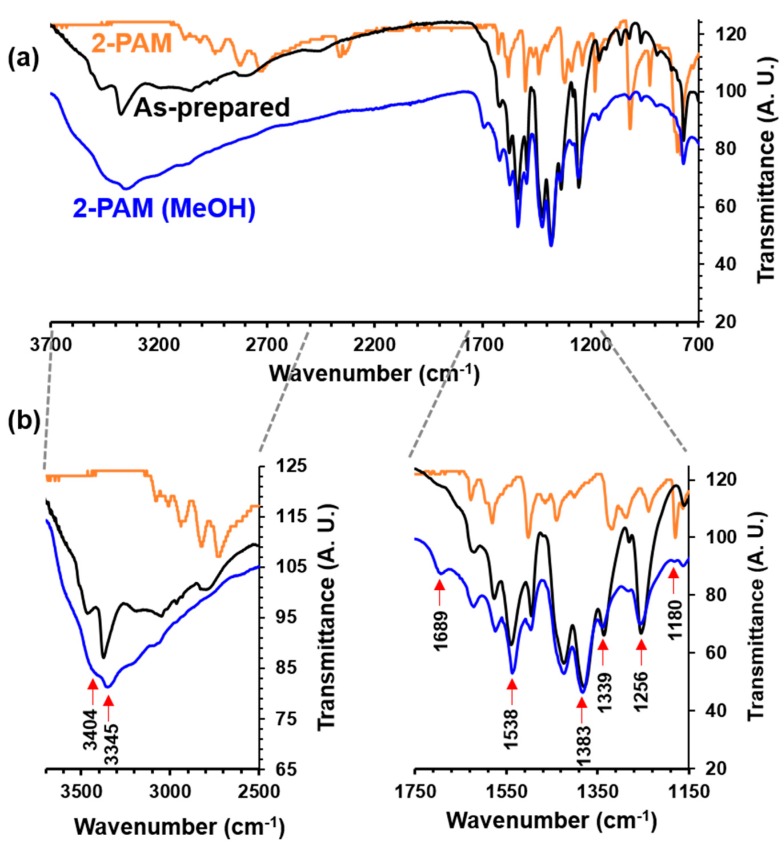
(**a**) FTIR spectra after drying at 100 °C for 2 h of the MIL-125-NH_2_ NPs, 2-PAM@MIL-125-NH_2_ and the free 2-PAM drug; and (**b**) Selected spectral regions highlighting the main differences between MIL-125-NH_2_ and 2-PAM@MIL-125-NH_2_.

After encapsulation, the reduction of BET surface area and pore volume values (1400 vs. 820 m^2^ g^−1^ and 0.55 vs. 0.34 cm^3^ g^−1^; Figure S8) confirmed the location of 2-PAM in the pores of the MIL-125-NH_2_ NPs. In addition, the pore size distribution is shifted to smaller sizes as a consequence of the drug encapsulation within the porosity (see Figure S9 in the ESI). The encapsulation of 2-PAM into the pores of MIL-125-NH_2_ NPs was also verified by FTIR studies (Figure 3) performed on dried samples (100 °C for 2 h). In the 1750–1150 cm^−1^ spectral range, the presence of the drug was deduced from: (i) the slight shift of the vibrational bands of MIL-125-NH_2_ centered from ca. 1546, 1379, 1336 and 1255 cm^−1^ to 1538, 1383, 1339 and 1256 cm^−1^, respectively; and (ii) an additional band located at ca. 1180 cm^−1^, characteristic of 2-PAM, only visible after encapsulation. In addition, a vibration mode appeared at ca. 1689 cm^−1^, which was attributed to a free carboxylic acid group, suggesting a partial degradation during the encapsulation process. As aforementioned, in the second selected spectral range, from 3700 to 2500 cm^−1^, two bands at ca. 3458 and 3384 cm^−1^ with medium and strong intensity, respectively, were assigned to the stretching υ_asym_(N–H) and υ_sym_(N–H) vibration modes of the –NH_2_ groups of MIL-125-NH_2_ solid. The same vibration bands were also observed for 2-PAM@MIL-125-NH_2_, however, υ_asym_(N–H) and υ_sym_(N–H) vibrations were shifted to ca. 3404 and 3345 cm^−1^, respectively (i.e., a less energetic region when compared with those assigned to the empty NPs). This suggests that 2-PAM molecules interact with the amino group of MIL-125-NH_2_ through the formation of hydrogen bonds.

**Figure 4:**
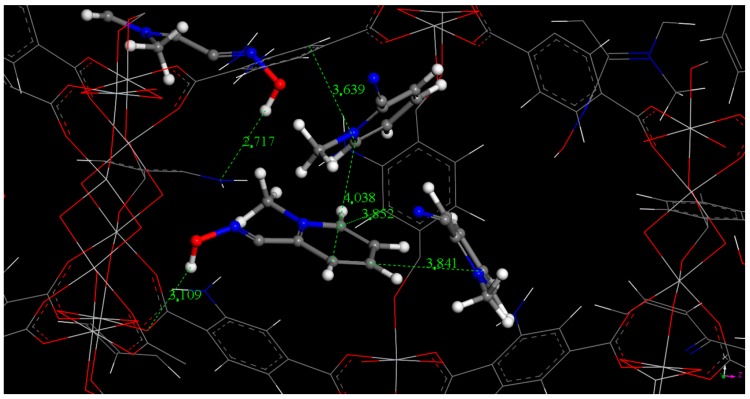
Main interaction sites existing between 2-PAM and MIL-125-NH_2_ framework from GCMC simulations: (white atoms: H, blue atoms: N, red atoms: O, clear grey atoms: Ti, dark grey atoms: C; for the sake of clarity Cl atoms are not shown).

The main interactions between 2-PAM and MIL-125-NH_2_ can be further investigated from statistical configurations extracted from GCMC simulations. Figure 4 illustrates π-stacking interactions between 2-PAM species and the phenyl ring of the solid, but also between 2-PAM molecules. In addition, the –NH_2_ groups from MIL-125-NH_2_, might establish specific hydrogen bonds with the hydroxyl groups of the 2-PAM (2.717 Å), in agreement with the FTIR observations. Finally, the –OH group of 2-PAM seems to reinforce the interaction between the drug and MIL-125-NH_2_ by interacting with the O coming from the carboxylate group of the MOF (3.109 Å). These observations suggest that, in agreement with TGA whereas only 1/3 of the theoretical maximun 2-PAM loading was achieved experimentally, only the 2-PAM molecules with the strongest interactions with the framework (see the distances MOF-NH_2_---HO-2-PAM (2.717 Å) and/or 2-PAM-OH---OOC-MOF (3.109 Å)), would be successfully encapsulated into MIL-125-NH_2_.

### 2.3. Colloidal Stability of 2-PAM@MIL-125-NH_2_

The presence of electrolytes in the physiological medium influences the colloidal stability of NPs and thus, their in vivo fate. Envisaging intravenous administration, MIL-125-NH_2_ and 2-PAM@MIL-125-NH_2_ NPs were dispersed in simulated biological fluids at 37 °C under continuous stirring (see Materials and Methods for further details): (i) a phosphate buffered saline solution (PBS) with electrolytes commonly present in serum (138 mM NaCl, 2.74 mM KCl and 10.0 mM Na_2_HPO_4_/NaH_2_PO_4_, pH = 7.4) and (ii) PBS supplemented with 10% *v*/*v* of heat deactivated fetal bovine serum (FBS), containing different macromolecules naturally present in the serum (proteins = 3.0–4.5 g/dL). Particle size and ζ-potential were monitored over a period of 24 h.

Despite the previously observed aggregation in aqueous solution, MIL-125-NH_2_ and 2-PAM@MIL-125-NH_2_ NPs in FBS-PBS exhibit a small and monodispersed particle size (ca. 220 nm), compatible with intravenous administration (a priori avoiding emboli phenomena) and comparable with those observed for the alcoholic solutions (Table 1). In the presence of phosphates, the ζ-potential becomes more negative when compared with pure water (−23 and −18 mV vs. −7 mV for MIL-125-NH_2_ and 2-PAM@MIL-125-NH_2_ NPs, respectively), in agreement with the attachment of phosphate groups on the outer NPs surface. To evaluate the influence of the ionic strength on the mobility measurements, ζ-potential was determined at different PBS concentrations (1 and 10 mM). More negative ζ-potential values were observed at lower ionic strength (−39 ± 8 vs. −23 ± 5 mV, respectively), however, with no significant differences on the particle size (170 ± 50 vs. 230 ± 60 nm, respectively).

Addition of FBS to the PBS resulted in a clear reduction on the ζ-potential magnitude (from −23 to −10 mV and from −18 to −9 mV for MIL-125-NH_2_ and 2-PAM@MIL-125-NH_2_ NPs, respectively), suggesting the formation of a protein corona onto the surface of both NPs [48]. As previously reported for other MOFs [49], the most abundant serum protein, albumin will be negatively charged at pH = 7.4 (isoelectric point = 4.7), replacing some phosphate groups at the outer surface and leading to the formation of a protein corona that might stabilize the MIL-125-NH_2_ colloidal solution by steric hindrance, instead of the electrostatic repulsions.

**Figure 5:**
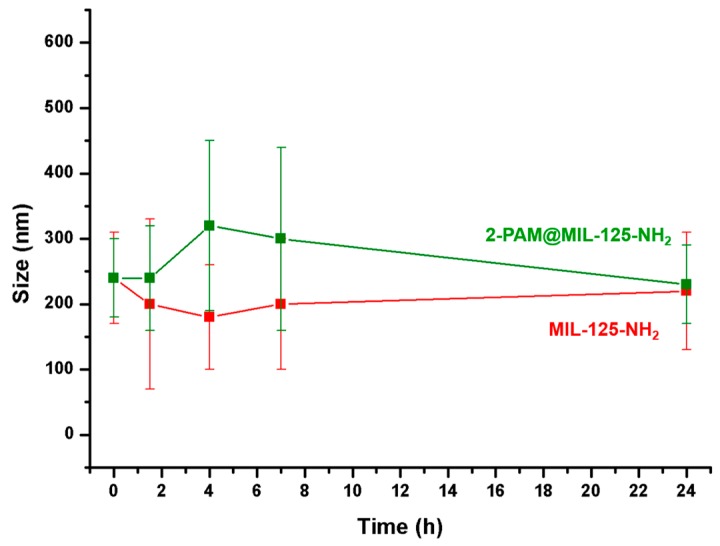
Variation of the particle size of MIL-125-NH_2_ and 2-PAM@MIL-125-NH_2_ in fetal bovine serum (FBS) supplemented with phosphate buffered saline (PBS) as a function of time.

The long-term colloidal stability of MIL-125-NH_2_ and 2-PAM@MIL-125-NH_2_ NPs was further monitored in FBS-PBS. No significant aggregation was observed for both NPs, which remained stable for 24 h (Figure 5). Remarkably, the absence of any 2-PAM@MIL-125-NH_2_ NPs aggregation in serum conditions is a required condition to avoid toxicity and support the crossing of the BBB. However, it is known that this passage is restricted when the size of the nanocarrier exceeds 100 nm [50]. Thus, a further reduction of the particle size of 2-PAM@MIL-125-NH_2_ NPs would be required to target the brain.

The stability during the storage of MIL-125-NH_2_ and 2-PAM@MIL-125-NH_2_ NPs was evaluated by simply drying the NPs at room temperature. Note that the dried NPs were kept for 6 months at room temperature and, after reconstitution in FBS-PBS and redispersion by sonication, their crystalline structure, particle size and superficial charge remained almost unchanged (for MIL-125-NH_2_: before and after storage: 210 ± 100 vs. 270 ± 70 nm and for PAM@MIL-125-NH_2_ NPs before and after storage: 240 ± 60 vs. 240 ± 40 nm; with ζ-potential values around −10 mV). These results confirm the feasibility of storing the 2-PAM@MIL-125-NH_2_ NPs for a prolonged period of time.

### 2.4. Release Kinetics of 2-PAM 

**Figure 6:**
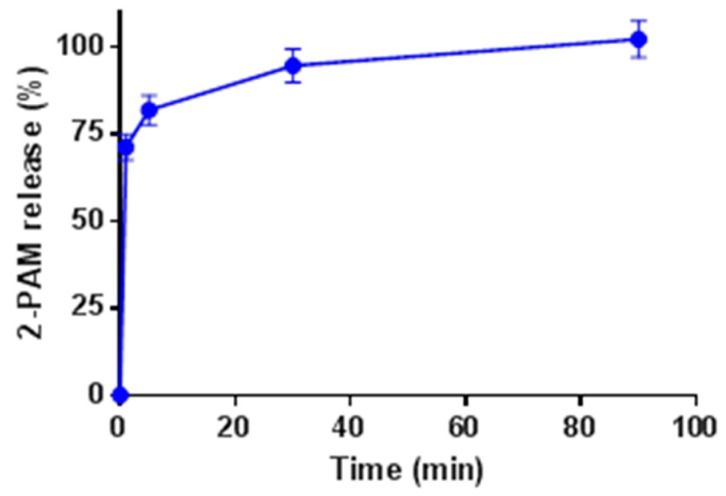
Release of 2-PAM from MIL-125-NH_2_ in PBS at 37 °C as a function of time. The concentration at 90 min was 240 μg mL^−1^. The error bars represent the coefficient of variation on the measurement.

The release kinetics of 2-PAM from the NPs was then analyzed, as 2-PAM needs to be in solution to exert its pharmacological effects. This was evaluated by suspending the 2-PAM@MIL-125-NH_2_ NPs in a simple in vitro medium (i.e., PBS) and monitoring the 2-PAM release over time by capillary electrophoresis (Figure 6; and see Materials and Methods for further details). During the first 5 min, the rapid release of ca. 82% of 2-PAM was observed. In the second stage, the remaining 2-PAM was progressively delivered in approximately 50 min. The first release step might be attributed to the 2-PAM molecules with the weaker hydrogen bond interactions (2-PAM-OH---OOC-MOF, 3.109 Å) between the drug and the MOF structure, whereas the second stage would correspond to release of the more strongly interacting species (MOF-NH_2_---HO-2-PAM, 2.717 Å), in accordance with the experimental and simulated studies (see Section 2.2). Additionally, it is likely that phosphates from the PBS medium replace 2-PAM molecules, as well as rapidly destroy the MOF framework to produce Ti phosphates, leading finally to an accelerated release. However, in contact with biological fluids, the formation of a protein corona might slow down the 2-PAM release, as already observed during in vivo studies for the MIL-100(Fe) NPs loaded with an antitumoral drug [51]. 

## 3. Materials and Methods 

### 3.1. General Instrumentation

X-ray powder diffraction (XRPD) of the MIL-125-NH_2_ nanoparticles were collected in an Empyream PANALYTICAL diffractometer (PANalytical, Lelyweg, The Netherlands), equipped with a PIXcel3D detector and with a copper radiation source (Cu Kα, λ =1.5406 Å), operating at 45 kV and 40 mA. XRPD of the 2-PAM-encapsulated MOF were collected in a Siemens D5000 (θ–2θ) diffractometer with Cu Kα1 radiation (λ = 1.54056 Å) from 3 to 30° (2θ) using a step size of 0.02° and 4 s per step in continuous mode. 

Fourier transform infrared (FTIR) spectra were collected using a Nicolet 6700 instrument (Thermo Scientific, Waltham, MA, USA) from 4000 to 400 cm^−1^. 

Ar sorption isotherms were obtained at 87 K using a Quadrasorb system (Quantachrome instruments, Boynton Beach, FL, USA). Prior to the analysis, approximately 30 mg of sample was evacuated at 150 °C under primary vacuum for 3 h. 

Thermogravimetric analyses (TGA) of the MIL-125-NH_2_ nanoparticles were performed using a SDT Q-600 thermobalance (TA instruments, New Castle, DE, USA) under air flow with a heating rate of 5 °C min^−1^. TGA of the 2-PAM@MIL-125-NH_2_ (5−10 mg) were recorded on a PerkinElmer Diamond TGA/DTA STA 6000 (PerkinElmer, Boston, MA, USA) under O_2_ atmosphere (20 mL min^−1^), at a heating speed of 3 °C min^−1^ for the temperature range between RT and 600 °C.

Field emission guns-scanning electron microscopy (FEG-SEM) images were acquired in a FEI/Philips XL-30 Field Emission ESEM (Philips, Amsterdam, The Netherlands) at 3 kV and 98–102 μA.

### 3.2. Reagents and Solvents

2-Aminoterephthalic acid (99%, Acros Organics, Thermo Fisher Scientific, Waltham, MA, USA), titanium isopropoxide (97%, Sigma Aldrich Co., St. Louis, MO, USA), phosphate buffered saline (PBS) solution (pH = 7.4, 0.01 M, Sigma Aldrich Co., St. Louis, MO, USA), heat-inactivated fetal bovine serum (FBS, Thermo Fisher Scientific, Waltham, MA, USA), pralidoxime (Sigma Aldrich Co., St. Louis, MO, USA), *N*,*N*’-Dimethylformamide (DMF, 99.5%, Chem-lab, Zedelgem, Belgium), methanol (analytical grade, J. T. Baker, Whitehouse Station, NJ, USA). All reagents and solvents were used as received from the commercial suppliers without further purification.

### 3.3. Synthesis of MIL-125-NH_2_ Nanoparticles

The nano-sized MIL-125-NH_2_ was isolated according to the following procedure: 1.375 g of 2-aminoterephthalic acid was dispersed in 20 mL of DMF and 5 mL of methanol in a round-bottom flask at room temperature. The mixture was stirred for a few minutes and heated at 100 °C. Finally, 1.5 mL of titanium isopropoxide and 0.09 mL of water were added and the resulting reactive mixture was kept at 100 °C until a color change (from orange to bright yellow) for 32 h. The obtained yellow solid was recovered by centrifugation (13,000 rpm for 5 min) and washed with DMF. The solid was then suspended in 100 mL of DMF for 3 h. Then, the solid was recovered by centrifugation and washed with 50 mL of methanol. Yield: 90%.

### 3.4. Encapsulation of 2-PAM into the Nano-Sized MIL-125-NH_2_

2-PAM was entrapped into the pores of MIL-125-NH_2_ by suspending 10 mg of the powdered MOF in 2.5 mL of a 16 mg mL^−1^ drug solution (in methanol), at room temperature under magnetic stirring for 8 h. The material/drug weight ratio was therefore 1:4. The 2-PAM-containing solid (2-PAM@MIL-125-NH_2_) was recovered by centrifugation and dried under air at room temperature.

### 3.5. Computational Simulation of 2-PAM Encapsulation

Using Grand Canonical Monte Carlo (GCMC) calculations, the saturation was estimated for 2-PAM in MIL-125-NH_2_ nanoparticles. It is thus possible to estimate the saturation capacity in the pores for the selected solids, as well as to elucidate the preferential adsorption sites for guest molecules on the solid framework. Partial charges and force field parameters were required for GCMC calculations. Simulations were performed at 300 K with a simulation box large enough to use a cut-off equal to 12.5 Å with typically 5 × 10^6^ Monte Carlo steps for both equilibration and prediction steps. In our calculations, saturation was considered to be reached with *P* = 10000 kPa.

Short range interactions were estimated using a cut-off distance of 12.5 Å. The differential adsorption enthalpy at low coverage was calculated through the fluctuation over the number of particles in the system and from the internal energy. 

The crystallographic structure considered for this study originated from the literature and has been optimized using force-fields with Forcite. In the GCMC calculations, the solid was supposed to be rigid and the unit cell parameters were therefore fixed at the experimental values. 

The adsorbed 2-PAM molecule was optimized by Density Functional Theory (DFT) calculations and considered in the GCMC calculations as rigid. It follows that no rearrangement of the conformation for the confined molecules was possible in the MIL-125-NH_2_ pores.

Regarding the solid, we used the Universal Forcefield (UFF) for Lennard Jones interatomic potentials, which is usually applied to describe solid interactions [52] and Equalization Electronegativity Method implemented in Material Studio to determine the partial charges distribution for the solid [53]. In contrast, the UFF parameters and DFT partial charges (calculated with a geometry optimization performed with DMol^3^ using PW91 functional and DNP basis set and high convergence criteria) were used for the adsorbate molecules.

### 3.6. Colloidal Stability of MIL-125-NH_2_ nanoparticles and 2-PAM@MIL-125-NH_2_

Particle size was monitored by dynamic light scattering (DLS) on a Zetasizer Nano ZS (Malvern Instruments). The following equation was used for the calculation of the polydispersity index: PdI = (width/mean)^2^. Nanoparticles were washed with the selected solvents (DMF, methanol, ethanol and water) to exchange the solvent into the pores, centrifuged (13,400 rpm, 7 min), redispersed in fresh solvent and sonicated for 30 s. This procedure was repeated 3 times. When water was used as solvent, the stability of MIL-125-NH_2_ nanoparticles was assessed in the 2–10 pH range. The pH was adjusted with NaOH 0.1 M and HCl 0.1 M solutions. Finally, particle size of the previously water-exchanged MIL-125-NH_2_ nanoparticles was also determined under simulated in vivo conditions using PBS and FBS supplemented FBS media. The solution of PBS with FBS was in situ prepared by adding 10% *v*/*v* of FBS to the previously prepared PBS solution. The final medium was sonicated for 30 s. The aggregation kinetics of MIL-125-NH_2_ nanoparticles was investigated by dispersing the nanoparticles in the selected media (at 20 °C) at concentration of 0.1 mg mL^−1^ by using an ultrasound tip at 20% amplitude for 20 s (UP400S, Hilscher, Teltow, Germany). Nanoparticles were weighed out (based on the wet/dry ratio previously determined from nanoparticles dried at 100 °C overnight). 

In parallel experiments, the particle surface charge was monitored by registering the ζ-potential of the colloidal solution at different times on a Zetasizer Nano instrument (Malvern, Worcestershire, UK). This value is obtained by measuring the electrophoretic mobility of the nanoparticles in dispersion by the electrophoretic light scattering (ELS) technique. 

The colloidal stability of the nanoparticles in the selected FBS-supplemented PBS medium was further evaluated over a period of 24 h. For these tests, 1 mg of nanoparticles was dispersed in 10 mL of the FBS supplemented PBS at the desired concentration and kept under bi-dimensional stirring at 37 °C. The particle size of MIL-125-NH_2_ and 2-PAM@MIL-125-NH_2_ was measured at different times (0, 1.5, 4, 7 and 24 h).

In order to validate the ζ-potential measurements in high ionic strength media such as PBS and PBS FBS, the experiments were also carried out under reduced voltage. The ζ-potential values for MIL-125-NH_2_ NPs when suspended in PBS were −23 ± 5, −21.3 ± 1 and 23.8 ± 0.4 mV using 50, 20 and 10 mV, respectively. In PBS FBS, the obtained ζ-potential values were −10 ± 4 mV, −10.9 ± 1 mV and −10.7 ± 2 mV applying a voltage of 50, 20 and 10 mV, respectively. Note here that 50 V were automatically selected by the equipment according with the analysis cell, the nature of the medium and the sample conductivity. These results demonstrate that voltage variation did not provoke blackening of the electrode or MOF degradation occurs due to the variation of the joule heating, supporting the validity of the obtained ζ-potential data.

### 3.7. 2-PAM Release Kinetics

2-PAM@MIL-125-NH_2_ (3.5 mg, loading 10.9% *w*/*w*) was loaded in a 4 mL Eppendorf vial. PBS buffer (1.5 mL) was added and the mixture was agitated by vortexing. At several time-points the mixture was centrifuged briefly and an aliquot (30 μL) was taken from the reaction mixture. The remaining mixture was vortexed again. The aliquot was transferred into a capillary electrophoresis (CE) sample vial and analyzed using CE. 

CE experiments were performed on a P/ACE System MDQ capillary electrophoresis instrument (Beckman Coulter, Fullerton, CA, USA) equipped with a Diode Array Detector (Beckman Coulter, Fullerton, CA, USA). A bare fused silica capillary (i.d. 75 μm, total length 60 cm, length to detector 50 cm) was mounted. Running buffer consisted of a 200 mM 6-aminohexanoic acid/acetic acid buffer, pH 4.55. The buffer was prepared by dissolving 2.6 g of 6-aminohexanoic acid and 1.14 mL acetic acid in 100 mL of water. The sample was introduced using a pressure injection for 5 s at 0.5 psi. Separation voltage was 15 kV. Detection was performed by measuring UV absorbance at 292 nm. 

The peak areas of 2-PAM were taken from the electropherograms and the corresponding 2-PAM concentrations were calculated from a calibration curve (slope 2.05 × 10^6^ AU.mL mg^−1^, *r*^2^ > 0.99; see Figure S11). The concentrations of 2-PAM determined at *t* = 90 min (0.25 mg mL^−1^) and *t* = 7200 min (0.24 mg mL^−1^, data not shown) were averaged and normalized to 100% release of 2-PAM from MIL-125-MH_2_. As such, the loading of the MOF was determined to be 10.3% *w*/*w* confirming the results by TGA (10.9%).

## 4. Conclusions

This manuscript describes the successful preparation of high yields of monodispersed MIL-125-NH_2_ nanoparticles (~220 nm) by using a simple, safe and low-cost methodology. These colloidally stable MIL-125 NH_2_ solutions were able to effectively encapsulate the nerve agent antidote 2-PAM into the MOF pores via π-stacking and hydrogen bond interactions. Further, both MIL-125-NH_2_ and 2-PAM@MIL-125-NH_2_ NPs exhibited high in vitro colloidal stability during 24 h. Despite the rapid release of 2-PAM under in vitro conditions, one could expect a more progressive drug delivery under in vivo conditions due to the formation of a protein corona, as suggested by our surface studies. In addition, further research could improve control over the drug delivery kinetics (e.g., surface grafting, MOF functionalization, other MOF topologies), thus improving the 2-PAM half-life. Given the simplicity of preparing MIL-125-NH_2_ nanoparticles and its remarkable colloidal stability, this nano-MOF system seems to be a powerful tool for application in biomedicine and other relevant fields requiring stable colloidal solutions.

## Figures and Tables

**Table 1:** Particle size and ζ-potential (with standard deviation) of the MIL-125-NH_2_ non-toxic nanoparticles (NPs) in different media at 20 °C.

NPs	Medium	Size (nm)	PdI	ζ-Potential (mV)
MIL-125-NH_2_	DMF	240 ± 70	0.16 ± 0.01	*
	Water	660 ± 250	0.263 ± 0.003	−7 ± 4
	Methanol	320 ± 100	0.10 ± 0.02	−40 ± 7
	Ethanol	220 ± 60	0.06 ± 0.03	−47 ± 19
	PBS	230 ± 60	0.18 ± 0.03	−23 ± 5
	PBS FBS	210 ± 100	0.220 ± 0.007	−10 ± 4
2-PAM@MIL-125-NH_2_	Methanol	630 ± 120	0.5 ± 0.1	−10 ± 7
	PBS	290 ± 130	0.3 ± 0.1	−18 ± 4
	PBS FBS	240 ± 40	0.4 ± 0.1	−9 ± 5

* Note here the impossibility of determining ζ-potential in adapted plastic cuvettes.

## Supplementary Materials

The following are available online at http://www.mdpi.com/2079-4991/7/10/321/s1: complementary PXRD, TGA, FTIR and SEM images of MIL-125-NH_2_ and 2-PAM@ MIL-125-NH_2_ NPs, and standard calibration curve of 2-PAM.
