# Supplementary material for: Nanometric MIL-125-NH2 Metal–Organic Framework as a Potential Nerve Agent Antidote Carrier
Source: Nanomaterials (Basel). 2017 Oct 12;7(10):321. doi: 10.3390/nano7100321 (PMC5666486; doi:10.3390/nano7100321)
Supplement: Supplementary file 1 [file nanomaterials-07-00321-s001.docx]

Supplementary Nanomaterials: Nanometric MIL-125-NH_2_ Metal-Organic Framework as Potential Nerve Agent Antidote Carrier

Sérgio M. F. Vilela ^1,†^, Pablo Salcedo-Abraira ^1,†^, Isabelle Colinet ^2^, Fabrice Salles ^3^,
Martijn C. de Koning ^4^, Marloes J. A. Joosen ^4^, Christian Serre ^2,5^ and Patricia Horcajada ^1,2^*

**Figure S1.** PXRD patterns of the simulated (bottom) and the nano-sized (top) MIL-125-NH_2_.

**Figure S2.** FEG-SEM images of the nano-sized MIL-125-NH_2_ before (left) and after (right) the 2-PAM encapsulation.


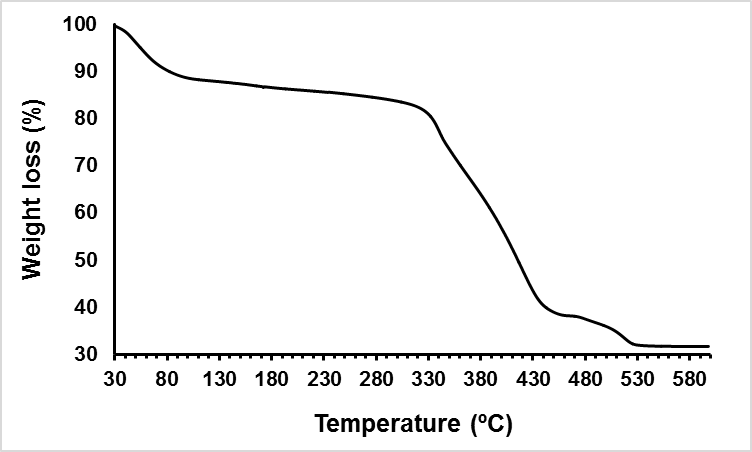


**Figure S3.** Thermogravimetric curve of the nano-sized MIL-125-NH_2_.

**Figure S4.** FTIR spectrum of the nano-sized MIL-125-NH_2_.


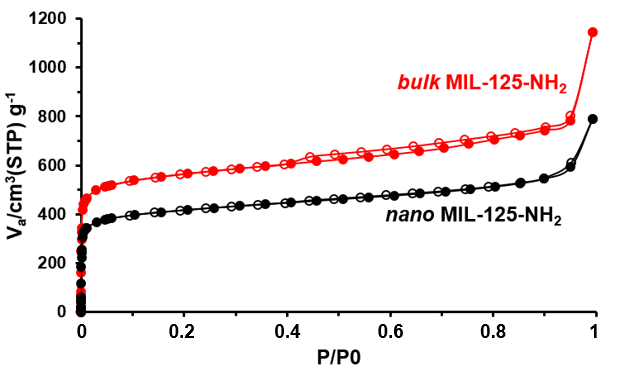


**Figure S5.** Ar adsorption isotherm of the *bulk* (**red**) and *nano* (**black**) MIL-125-NH_2_ measured at 87 K.


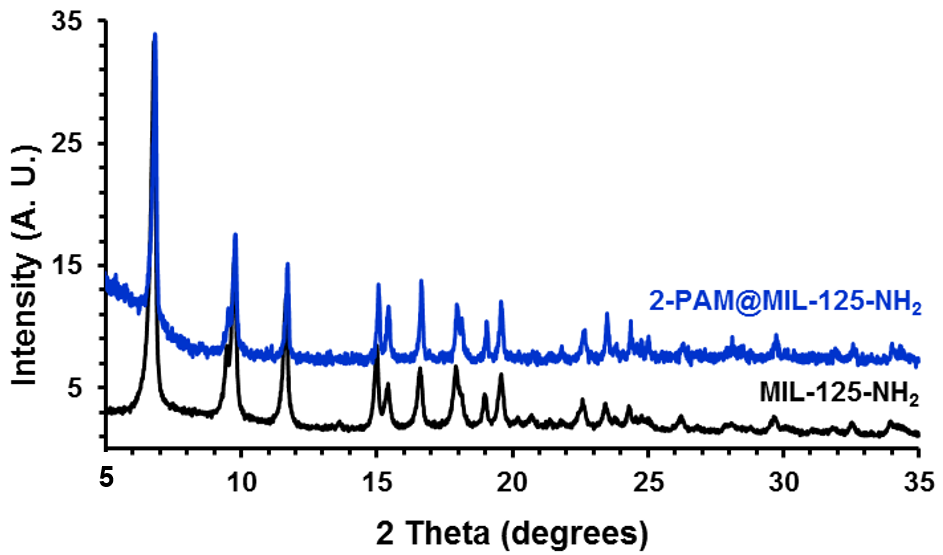


**Figure S6.** PXRD patterns of MIL-125-NH_2_ and 2-PAM@MIL-125-NH_2_.


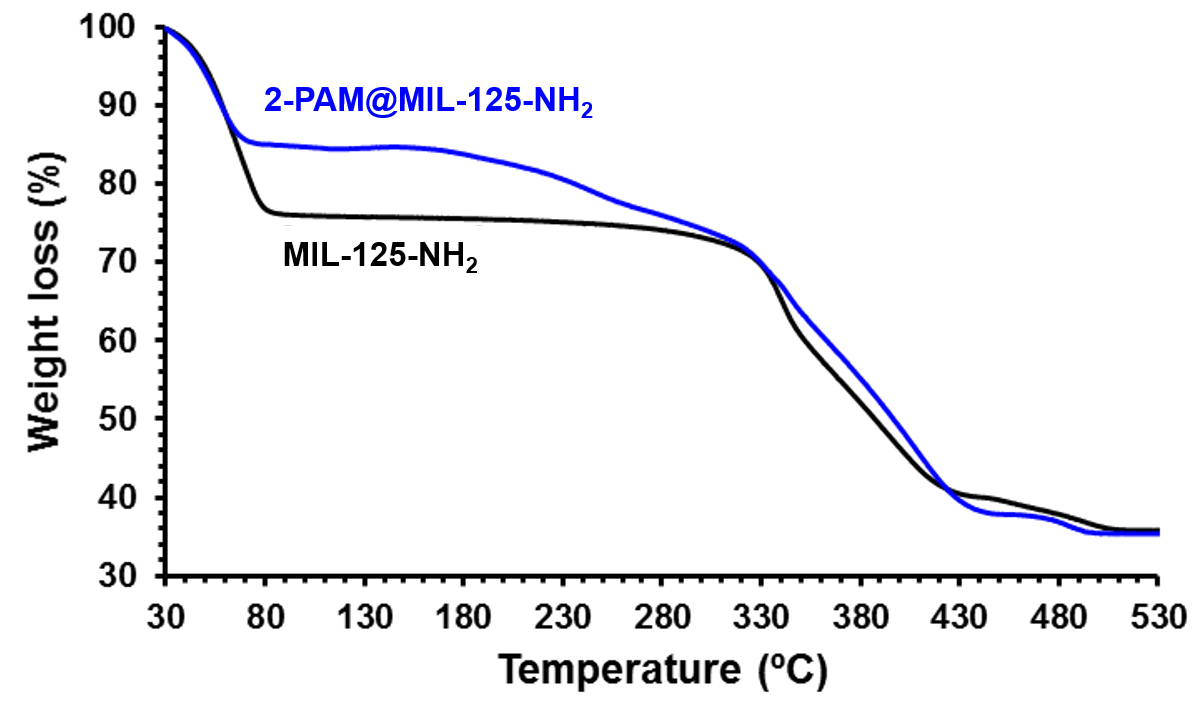


**Figure S7.** TGA curves of MIL-125-NH_2_ and 2-PAM@MIL-125-NH_2_.


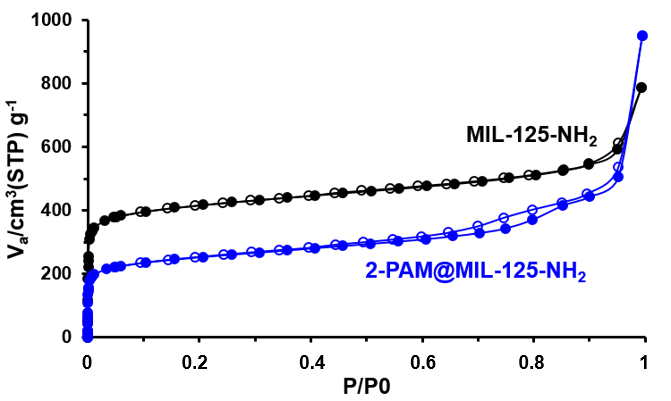


**Figure S8.** Ar adsorption isotherm of the MIL-125-NH_2_ nanoparticles before (**black**) and after (**blue**) encapsulation of 2-PAM, measured at 87 K.


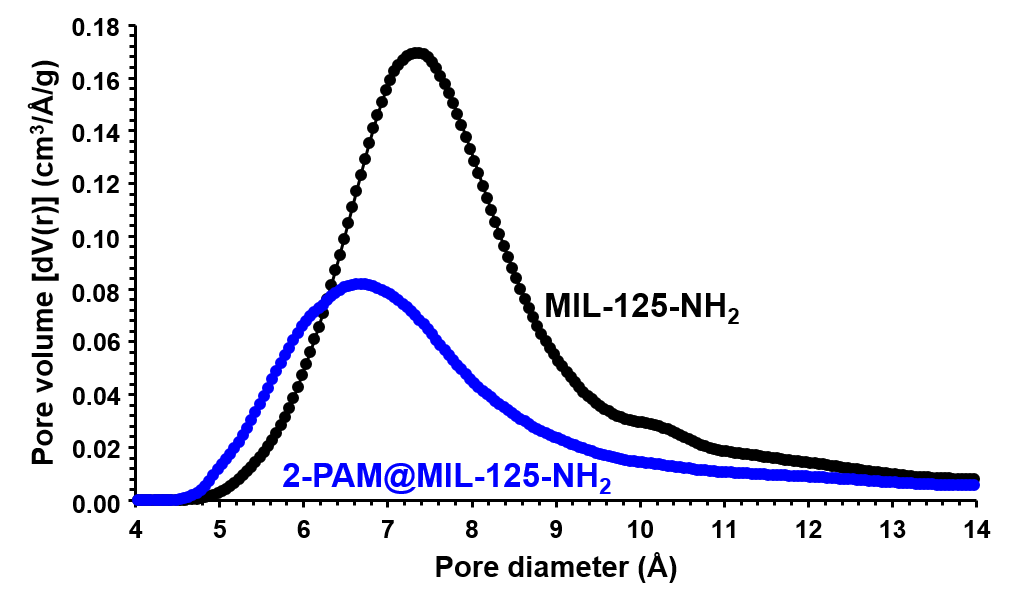


**Figure S9.** Horvath-Karazoe pore size distribution of MIL-125-NH_2_ nanoparticles before (**black**) and after (**blue**) encapsulation of 2-PAM, measured experimentally at 87 K using Ar.


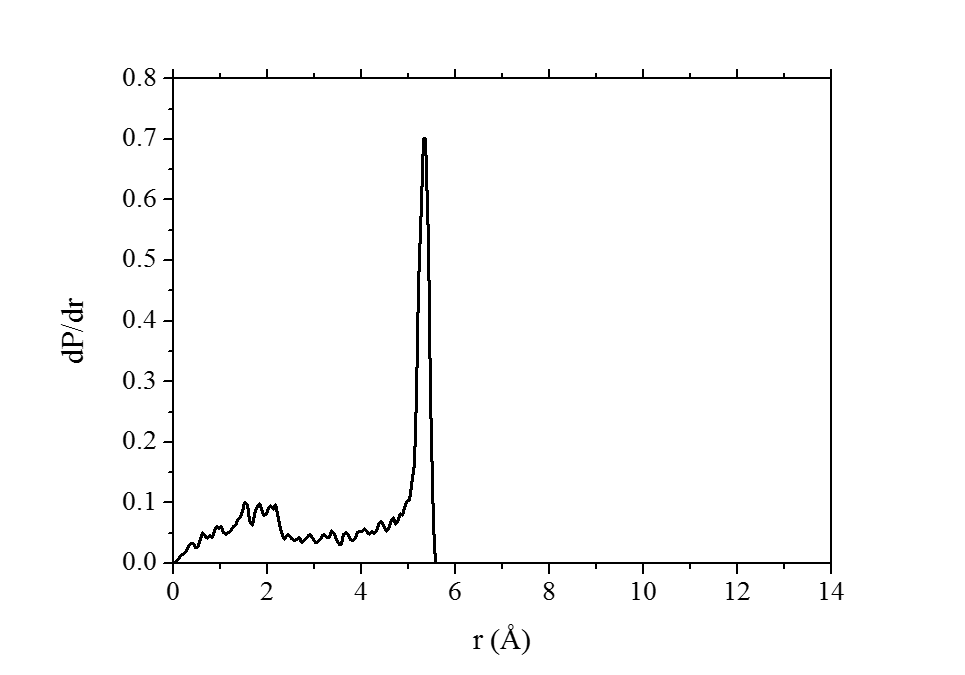


**Figure S10.** Pore size distribution for MIL-125-NH_2_ obtained from theoretical calculations.

The pore size distribution reported in Figure S10 illustrates the monodisperse distribution of the pore size in MIL-125-NH_2_. The pore sizes are estimated at 2 and 5.5 Å using the strategy developed by Gelb and Gubbins [1] and UFF force field. From the pore size distribution, it is possible to estimate the cavity sizes (4 and 11 Å), close to the estimated sizes extracted from crystallographic data.

**Figure S11.** Standard calibration curve of 2-PAM.

**References**

[1] Gelb, L.D.; Gubbins, K.E. Distribution in Porous Glasses: A Computer Simulation Study. *Langmuir* **1999**, *15*, 305−308.
